# Supplementary material for: Acceptance of Social Networking Sites by Older People before and after COVID-19 Confinement: A Repeated Cross-Sectional Study in Chile, Using the Theory of Planned Behaviour (TPB)
Source: Int J Environ Res Public Health. 2022 Oct 16;19(20):13355. doi: 10.3390/ijerph192013355 (PMC9602972; doi:10.3390/ijerph192013355)
Supplement: Supplementary file 1 [file ijerph-19-13355-s001.zip › ijerph-1925505-supplementary.pdf]

Table S1. Longitudinal and pseudo-longitudinal studies based on TPB.

Plotnikoff et al. (2012). Objective: To predict physical activity intention in a random sample of Canadian adults and examine whether gender moderates the relationships between TPB and physical activity intention.			
	**T1** (**1998**) (1427 subjects)	**T2** (**2003**) (1427 subjects)	**Differences MGA**
ATT > Intention	0.30***	0.22***	Not analysed
SN > Intention	0.11***	n/d	Not analysed
PBC > Intention	0.29***	0.11***	Not analysed
R^2	$R^2 = 0.29$	$R^2 = 0.21$	
Leung (2019). Objective: To examine how people's intentions change after they visit a Facebook page associated with tourist destinations.			
	**T1** (**base**) (297 subjects)	**T2** (**3 months after**) (172 subjects)	**Differences MGA**
ATT > Intention	n.s.	-0.15*	-0.214* (decreases at T2)
SN > Intention	0.25**	0.54**	0.293* (increases at T2)
PBC > Intention	0.21**	0.22*	0.008 (similar at both T1 and T2)
R^2	$R^2 = 0.24$	$R^2 = 0.42$	
Roux et al. (2021). Objective: To conduct a longitudinal study based on the TPB theory to explain the intention to engage in physical activity in basic education children in France and to examine whether gender moderates the relationships between TPB and the intention to engage in physical activity.			
	**T1** (**base**)	**T2** (**3 months after**)	**Differences MGA**
ATT > Intention	0.14**	0.14**	Not analysed
SN > Intention	0.21***	0.12**	Not analysed
PBC > Intention	0.34***	0.38***	Not analysed
R^2	$R^2 = 0.31$	$R^2 = 0.38$	
Thaker & Ganchoudhuri (2021). Objective: To identify changes in the intention to be inoculated with the Covid-19 vaccine in the New Zealand population.			
	**T1** (**March 2021**)	**T2** (**May 2021**)	**Differences MGA**
ATT > Intention	-0.31***	-0.16***	Not analysed
SN > Intention	-0.14***	0.02	Not analysed
PBC > Intention	Not analyzed	Not analyzed	Not analysed
R^2	$R^2 = 0.74$	$R^2 = 0.55$	
Liu et al. (2022) [1]. Objective: To develop a model based on TAM and TPB to predict the intention to use information technologies (tablets) in health in patients with chronic diseases in China.			
	**T1** (**baseline**)	**T2** (**24 weeks after**)	**Differences MGA**
ATT > Intention	0.03 (n.s.)	0.27**	B (8 weeks) > B (baseline) *<br>B (24 weeks) > B (baseline) *
SN > Intention	0.09 (n.s.)	0.08 (n.s.)	No significant
PBC > Intention	0.38**	0.19 (n.s.)	B (8 weeks) > B (24 weeks) *
R^2	$R^2 = 0.51$	$R^2 = 0.73$	

ATT: attitude; SN: subjective norms; PBC: perceived behavioural control; n/a: not measured; *p<0.05; **p<0.01; ***p<0.001;

[1] In this longitudinal study. data were additionally collected at 8 and 16 weeks.
